# Protective Effect of a Polyherbal Aqueous Extract Comprised of* Nigella sativa* (Seeds),* Hemidesmus indicus* (Roots), and* Smilax glabra* (Rhizome) on Bleomycin Induced Cytogenetic Damage in Human Lymphocytes

**DOI:** 10.1155/2017/1856713

**Published:** 2017-05-25

**Authors:** Bandula Prasanna Galhena, S. S. R. Samarakoon, Myrtle Ira Thabrew, Solomon F. D. Paul, Venkatachalam Perumal, Chinnadurai Mani

**Affiliations:** ^1^Department of Biochemistry and Clinical Chemistry, Faculty of Medicine, University of Kelaniya, Ragama, Sri Lanka; ^2^Institute of Biochemistry, Molecular Biology, and Biotechnology, University of Colombo, Colombo, Sri Lanka; ^3^Department of Human Genetics, Sri Ramachandra University, Chennai, India; ^4^Department of Oncologic Sciences, Mitchell Cancer Institute, University of South Alabama, Mobile, AL, USA

## Abstract

This study was carried out to determine the chemoprotective potential of a polyherbal aqueous decoction comprised of* Nigella sativa* (seeds),* Hemidesmus indicus* (roots), and* Smilax glabra* (rhizome) against bleomycin induced cytogenetic damage in human lymphocytes. Isolated peripheral blood lymphocytes (PBLs) were exposed to bleomycin at a dose of 40 *µ*g/mL for 2 hrs in the presence or absence of different doses of the decoction (100, 300, and 600 *µ*g/mL). Modulatory effect of the decoction on bleomycin induced cytogenetic damage was evaluated by (a) degree of chromosomal aberrations (CA), (b) formation of micronuclei (MN), and (c) induction of *γ*H2AX foci in lymphocytes exposed to bleomycin. Lymphocytes pretreated with the decoction showed that a significant reduction (*p* < 0.05) in bleomycin induced (a) stable and unstable chromosome aberrations (CA), (b) MN formation, and (c) formation of *γ*H2AX foci, when compared to lymphocytes treated only with bleomycin. The decoction by itself did not induce any significant cytogenetic damage in PBLs. Overall results of the present study confirm that the decoction can attenuate the cytogenetic damage mediated by bleomycin in human PBLs.

## 1. Introduction

Chemotherapy is one of the treatment modalities that arrests growth of malignancy either by killing transformed cells or by arresting their division. Chemotherapy is often used either as adjuvant or neoadjuvant or in conjunction with radiotherapy and surgery. However, the major drawback of using such cancer therapeutics is that it may lead to transient and/or permanent damage to normal tissues due to their direct toxicity to healthy cells or toxicity mediated by signals generated by target cancer cells [1, 2]. Nausea, vomiting, diarrhoea, inflammation of the intestinal and oral mucosa, loss of appetite, fatigue, anaemia, leukopenia, pain, and initiation of secondary neoplastic changes are some of the common symptoms of toxicities during cancer chemotherapy [3–6]. Generation of free radicals and initiation of uncoordinated inflammatory response are some of the principle mechanisms of toxicities derived from cancer chemotherapy or radiotherapy [7–10]. Bleomycin (BLM), a commonly used anticancer drug, has been reported to exert free radical mediated toxic effects in similar manner to that of ionized radiation (IR) [11]. BLM induces DNA strand breaks, resulting in production of DNA adducts and excess reactive oxygen species (ROS) leading to oxidative stress, mitochondrial leakage, and apoptosis [12]. Bleomycin at a concentration of 40 *μ*g/mL can induce radiomimetic damage equal to 2 Gy of gamma radiation which is the standard fraction applied daily to cancer patients in fractionation regimes used in radiotherapy [13].

Natural antioxidants have been considered to be promising protective agents against free radical mediated damage induced during cancer therapy. A number of effective compounds such as hoechst [14], cysteine [15], caffeine [16], calcium channel blockers [17], flavonoids [18], and 2-deoxy-D-glucose [19] have been identified. Natural antioxidants have also demonstrated effective protection against ROS mediated cellular damage during cancer chemotherapy and radiotherapy [20–22]. However, there are comparatively few investigations that investigate the use of natural protectors during chemotherapy and/or radiotherapy. The importance of such protectors is emerging due to frequent use of chemotherapeutics in combinational therapy with radiation, causing higher magnitude of toxicity than either of the above two modes of therapy.

Polyherbal decoction comprised of* Nigella sativa* seeds,* Hemidesmus indicus* roots, and* Smilax glabra* rhizome in equal proportions has traditionally been used by a particular family of Ayurvedic physician in Sri Lanka to treat cancer patients [23]. Recent studies have demonstrated that the above decoction can provide significant protection against chemically induced hepatocarcinogenic changes in rats [24] without producing any significant toxic effects and exert cytotoxicity in human hepatoma (HepG2) cells [25]. Protection against oxidative damage [26], favourable immune modulation [27], and expression and suppression of proapoptotic and antiapoptotic genes [28] are some of the mechanisms which mediate anticancer activity of the decoction. It was hypothesized that the claimed antioxidant activity of the decoction may play a dual role: (a) protecting healthy cells against the damage mediated by either chemotherapy or radiotherapy and (b) restricting the tumour progression by resisting mutation in cancer management.

Therefore, the present study was carried out with the primary objective of evaluating the effectiveness of the decoction comprised of* N. sativa* seeds,* H. indicus* roots, and* S. glabra *rhizome, in protecting against bleomycin induced cytogenetic damage in peripheral blood lymphocytes (PBLs).

## 2. Materials and Methods

### 2.1. Plant Material

Seeds of* N. sativa *(Ranunculacaea), roots of* H. indicus* (Asclepiadaceae), and rhizome of* S. glabra *(Smilacaceae) were purchased and authenticated by the botanist at the Bandaranayaks Memorial Ayurvedic Research Institute (BMARI), Nawinna, Maharagama, Sri Lanka. Voucher specimens (UKFM/B/2006/01, UKFM/B/2006/02, and UKFM/B/2006/03) have been deposited at the Department of Biochemistry and Clinical Chemistry, Faculty of Medicine, University of Kelaniya, Sri Lanka.

### 2.2. Preparation of Standardized Decoction

Standardized decoction was prepared as previously described by Samarakoon et al. [28]. Equal portions (20 g each) of* N. sativa *(seeds),* H. indicus *(roots), and* S. glabra *(rhizome) were mixed and boiled gently in 1.6 L of distilled water until a final volume of 200 mL was obtained. The extract was then filtered, freeze dried, and stored in a vacuum desiccator at −4°C. The percentage yield of the freeze dried plant material was 13.6%. For experimental purposes, the required weight of the freeze dried powder was reconstituted in 1% dimethyl sulfoxide (DMSO) and filter sterilized using 0.22 *μ*m disposable filters.

### 2.3. Blood Sample Collection

Study was approved and carried out according to the guidelines of Ethical Review Committee, Sri Ramachandra University, Chennai, India. Heparinized blood samples (~10 mL) were obtained with informed consent and according to institutional procedures from six healthy donors aged 24–34 years, without any history of smoking, tobacco chewing, or alcohol consumption, and they were not taking any drugs for medical or other reasons.

### 2.4. Experimental Design

Out of 10 mL of blood collected from each individual, equal volume of 4 mL was used for chromosome aberration (CA) studies and cytokinesis-blocked micronuclei (CBMN) assay while the remaining blood was used for *γ*H2AX assay. For each CA and CBMN assays, 4 mL of blood was reconstituted with 36 mL of RPMI-1640 culture medium supplemented with 7.5% NaHCO_3_, 20% fetal calf serum, 200 mM L-glutamine, penicillin (100 units/mL), and streptomycin (100 *μ*g/mL) and equally aliquoted into eight sterile vials. Two sets of vials (each set consisting of three vials) were added with the decoction at concentrations of 100, 300, and 600 *μ*g/mL. Each set of vials served as decoction control and the test, respectively. All vials were then incubated at 37°C in 5% CO_2_ for 3 hrs. At the end of the 3rd hour, bleomycin at a concentration of 40 *μ*g/mL was added to (a) a one set of vials containing three different doses of the decoction and (b) a vial without the decoction (bleomycin control) and all were further incubated for 2 hrs. A vial with addition of neither decoction nor bleomycin served as negative control. After 5 hrs of incubation, cells in each vial were washed with Hanks Balanced Salt Solution (HBSS) three times. Each sample was then reconstituted in 4.5 mL of culture medium (RPMI-1640) and used for CA and CBMN analysis as described below.

For *γ*H2AX assay, remaining 2 mL of blood was reconstituted with 18 mL of RPMI-1640 culture medium and equally aliquoted into four sterile vials. Decoction at a concentration of 100 *μ*g/mL was then added to two vials (decoction control and test) and all vials were incubated at 37°C in 5% CO_2_ for 3 hrs. At the end of the 3rd hour, bleomycin at a concentration of 40 *μ*g/mL was added to (a) a vial containing the decoction (test) and (b) a vial without the decoction (positive control). Subsequently, all vials were incubated for further 2 hrs and lymphocytes were isolated by layering 4 mL aliquot from each vial on to 2 mL of Histopaque–1077 and centrifuged at 1000*g* for 5 min at room temperature.

### 2.5. Processing of Cultures for Chromosomal Aberration

Lymphocyte cultures for chromosomal aberration assay were processed as described earlier [29]. Briefly, cultures were initiated by adding 0.2 mL of phytohemagglutinin into each vial containing 0.5 mL of blood and 4.5 mL of RPMI-1640 supplemented with 7.5% NaHCO_3_, 20% fetal calf serum, 200 mM L-Glutamine, penicillin (100 units/mL), and streptomycin (100 *μ*g/mL) and incubated at 37°C in 5% CO_2_. At 46th hour of incubation, the cells were blocked at metaphase by adding colcemid at a final concentration of 0.1 *μ*g/mL and the cultures were further incubated until 48 hrs. The samples were then harvested after hypotonic treatment (20 minutes with 0.45% KCl at 37°C), washed three times with Carnoy's fixative, and cast on clean precooled slides. All slides were stained with 10% Giemsa, air-dried, and mounted with cover-slip using DPX mounting medium to analyze the chromosomal aberrations.

### 2.6. Processing of Cultures for CBMN Assay

Lymphocyte cultures for micronuclei assay were set up according to method described by Fenech and Morley [30]. Briefly, cultures were initiated as described in chromosomal aberration assay and at the end of 44th hour of culture Cytochalasin-B at a final concentration of 3 *μ*g/mL culture was added to each vial. The cells were further incubated for 28 hrs at 37°C. The cells were harvested with brief hypotonic treatment and slides were prepared by fixing the cells with Carnoy's fixative. The cell suspensions were dropped onto a clean cooled slide and stained with Giemsa.

### 2.7. Lymphocyte Preparation for *γ*H2AX Foci Assay

Isolated lymphocytes were spotted onto microscope slides (six slides/each individual) at an optimized cell density for immunofluorescence staining. Cells were fixed in 3.7% paraformaldehyde in PBS (15 min at room temperature) and permeabilised using 0.5% Triton X 100 in PBS (5 min at 4°C). Samples were blocked in phosphate buffered saline (PBS) with 2% bovine serum albumin (BSA) for 3 × 5 min at room temperature. Samples were then incubated with anti-*γ*H2AX antibody (clone JBW301) overnight at 4°C, and washed in PBS, 2% BSA, for 2 × 5 min. Finally slides were incubated with FITC-conjugated goat anti-mouse secondary antibody (Invitrogen, Paisley, UK) for 1 h at room temperature. Slides were washed in PBS for 2 × 2 min, stained with 4,6-diamidino-2-phenylindole (DAPI) for 5 min, washed for 2 min in PBS, and mounted using Mowiol Mounting Medium.

### 2.8. Slide Scoring

For cytogenetic analysis, preparations were coded and scored blind by two examiners. Chromosomal aberrations were analyzed in 100 metaphase spreads for each individual. Cells were analyzed for chromosome and chromatid types of aberrations as described earlier [31].

Scoring of MN was limited to binucleate (BL) lymphocytes only with preserved cytoplasm according to the criteria proposed by Fenech et al. [32]. Identification of binucleate cells in cell groups required careful visual examination of the individual cell boundaries. The results are expressed as number of micronucleated cells per 1000 binucleate cells on the two different slides from the same culture. Proliferation kinetics data was calculated by considering the frequencies of mono-, di-, tri-, and tetranucleate cells per each treatment group. A nuclear division index (NDI) was calculated according to the formula proposed by Takeshita et al. [33] as follows: NDI = (1*M*_1_ + 2*M*_2_ + 3*M*_3_ + 4*M*_4_)/*N*, where *M*_1_ to *M*_4_ represent the number of cells with one to four nuclei and *N* is the total number of cells scored.

For the viewing of *γ*-H2AX immunofluorescence foci, an epifluorescence microscope (ProvisAX70, Olympus) was used. Fields were initially selected at 10x on the basis of DAPI counterstained nuclei. Acquired DAPI image was used to define the focusing area and the detection of foci was performed using 40x objectives at optimized detection parameters. Scoring of foci was carried out manually in double blinded manner.

### 2.9. Statistical Analysis

From the various types of aberration recorded the aberration frequency was calculated as follows:

(1) Aberration  Frequency=Number  of  aberrations  observed  per  sampleNumber  of  metaphases  scored  per  sample.

The standard error for the aberration frequency was calculated as follows:

(2) Standard  error=Number  of  aberrations  observed  per  sampleNumber  of  metaphases  scored  per  sample.

Multiple comparisons between different experimental groups were done using multifactor ANOVA.

## 3. Results

### 3.1. Effects on Chromosomal Aberration

Dicentric chromosomes (DC), acentric fragments (AF), and chromatid breaks (ChB) were the indices used to evaluate the chromosomal aberrations during the present study. The results of unstable chromosomal aberrations in the four experimental groups are summarized in Table 1. The baseline chromosomal damage of the negative control was negligible (DC = 0.037, AF = 0.043, and ChB = 0.063) and was comparable to that in published literature. Further, it was observed that there was no significant change in chromosomal aberrations due to introduction of the decoction at the dose range of 100–600 *μ*g/mL.

Chromosomal aberrations in bleomycin control were significantly high when compared with the negative control. On exposure to bleomycin, there was a 7-fold increase in the frequency of cells with dicentrics (0.27 per 100 cells) when compared to the index in negative control group (0.037 per 100 cells). The increases observed in AF (8-fold increase) and ChB (7-fold increase) were similar to that observed with respect to DC. In the test cells (lymphocytes exposed to bleomycin plus the decoction) there was a significant reduction (*p* < 0.05) in chromosomal damage when compared with the bleomycin control. The reductions in DC, AF, and ChB at the decoction dose of 300 *μ*g/mL were nearly 2-fold that of the bleomycin control. The maximum percentage of inhibitions for DC (48%), AF (47%), and ChB (37%) was observed at the decoction dose of 300 *μ*g/mL. However, a slight deterioration in the protection was observed at a decoction dose of 600 *μ*g/mL (Table 1).

### 3.2. Effects on Cytokinesis-Blocked Micronuclei (CBMN) Formation

Prevalence of MN in four experimental groups is illustrated in Table 2. The mean MN frequency in control PBLs was 10 MN per 1000 binucleated cells. Similar to effect in chromosomal aberration study, the decoction by itself did not induce any excess MN. A slight statistically insignificant elevation in MN count was observed in PBLs exposed to the decoction (100–600 *μ*g/mL), with respect to the control samples. These results suggest that the decoction at a concentration range of 100–600 *μ*g/mL does not induce any significant damage to lymphocyte genome in vitro.

As expected nearly 9-fold increase in MN (89 MN per 1000 binucleated cells) was observed in lymphocytes exposed to bleomycin for 2 hrs at a dose of 40 *μ*g/mL (equal to that of 2 Gy of gamma radiation). This increase was consistent in all three subjects tested.

Bleomycin induced damage was significantly reduced (*p* < 0.05) in lymphocytes preexposed to the decoction at the tested dose range. Overall reduction was nearly 72% (25 MN per 1000 binucleated cells) when compared with the bleomycin control (88 MN per 1000 binucleated cells). This result suggests a significant protective effect of the decoction against genomic damage induced by bleomycin.

Nuclear division index (NDI) was calculated to assess the effects of the decoction on the proliferative capacity of lymphocytes. NDI in lymphocytes treated with the decoction is same as that of control, suggesting no interference of the decoction in human lymphocyte proliferation. However, a significant delay in lymphocyte proliferation in the presence of bleomycin at a concentration of 40 *μ*g/mL was observed. This was manifested by a change in relative numbers of *M*_1_ to *M*_4_ cells. However, restoration of NDI was observed in lymphocytes pretreated with the decoction prior to bleomycin induction. This reflects a successful overcome of cytotoxic effects caused by bleomycin, by modulating the lymphocyte proliferation.

### 3.3. Effects on Formation of *γ*H2AX Foci

**Figure 1:**
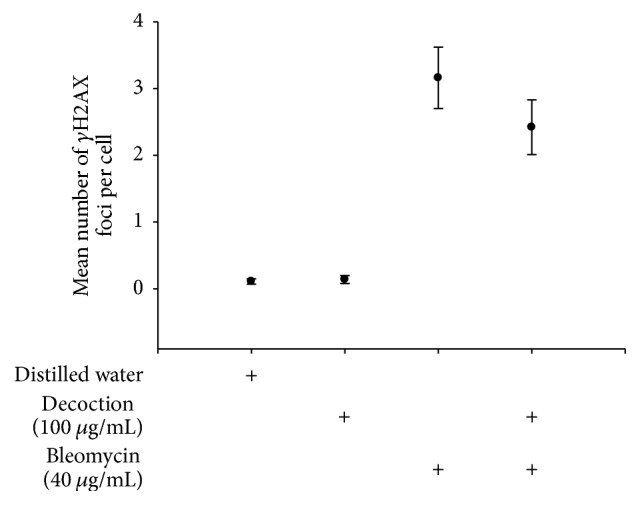
Mean number of *γ*-H2AX foci observed in lymphocytes obtained from four (4) experimental groups. Data represents mean ± SD of six samples from each experimental group.

The effect of the decoction on bleomycin induced *γ*-H2AX foci in peripheral lymphocytes is illustrated in Figure 1 and Table 3. Baseline DNA damage in the present study was at 0.11 ± 0.04 foci per cell, represented by 674 cells without a single foci and 218 and 108 cells with single and double foci, respectively. None of the control cells indicated more than two (2) foci within a cell. Decoction by itself (at a concentration of 100 *μ*g/mL) did not induce any significant DNA damage, as it indicated 0.14 ± 0.06 foci per cells with a distribution at 0–2 foci per cell. This observation is compatible with the previous results of MN and CA assays. Bleomycin (40 *μ*g/mL), an equal dose to 2 Gy irradiation, significantly induced *γ*-H2AX foci (3.16 ± 1.13), with a distribution range of 0–10 foci per cell. Out of 1000 cells analyzed, approximately 700 cells were bearing either 2, 3, or 4 foci per cell. This was compatible with previous reports on lymphocytes exposed to 2 Gy irradiation.

It was interesting to observe a significant reduction in *γ*-H2AX yield in lymphocytes exposed to bleomycin (40 *μ*g/mL) pretreated with the decoction at a dose of 100 *μ*g/mL. The number of foci per cell stood at 2.42 ± 1.01 foci within the range of 0–9 foci per cell. However, majority of cells (approx. 600 cells) indicated either 0, 1, or 2 foci per cell while 340 cells were reported to contain 3 foci per cell. The present results further confirm the ability of the decoction to protect against cytogenetic damage mediated by irradiation.

## 4. Discussion

A wide variety of chemotherapeutic agents are used routinely in clinical oncology. These therapies can also lead to cytotoxic and genotoxic effects in normal cells of the body, in addition to their effects on tumour cells [1, 3]. Any agent that could modulate such toxicity to normal cells would therefore be of much value in reducing the very unpleasant side effects experienced by cancer patients receiving chemotherapy.

It is well documented that bleomycin, like most other chemotherapeutic drugs, can mediate DNA changes in both cancer cells and normal cells. As with ionizing radiation, Bleomycin mediates its genotoxic effects by inducing DNA double strand breaks (dsbs) which is one of the critical lesions for cellular death [11]. This “radiomimetic” agent induces such double strand breaks by highly specific, concerted free radical attack on deoxyribose moieties in both DNA strands [33, 34]. Although the lesion induced by bleomycin is supposed to be only a small subset of the many lesions induced by ionizing radiation, effects of this radiomimetic agent in cells are remarkably similar to those of ionizing radiation [35].

The present investigation has shown that the decoction comprised of* N. sativa *seeds,* H. indicus* roots, and* S. glabra* rhizomes can significantly protect against bleomycin induced (a) chromosomal aberrations, (b) formation of micronuclei, and (c) DNA dsbs in normal human peripheral blood lymphocytes. Decoction itself induces no significant chromosomal aberrations, no micronuclei formation, nor DNA dsbs in lymphocytes.

Nuclear Division Index (NDI) is calculated to assess the effects of decoction on the proliferative capacity of human lymphocytes [36]. From NDI values calculated in the present investigation, it was apparent that bleomycin caused a delay in cell proliferation, manifested by a change in relative numbers of cells with one nucleus to cells with four nuclei (*M*1 to *M*4 cells). The NDI values in lymphocytes exposed to bleomycin alone are similar to those reported by other investigators [37]. In general, different types of ionizing radiation delay lymphocyte cell cycle and the extent of such delay depends on the dose delivered. An average mitotic delay of about 1 h per 1 Gy of gamma radiation has been observed once lymphocytes are exposed to ionizing radiation [38]. These delays in cell cycle allow the cell to repair DNA and try to reduce the adverse effects of the irradiation [39]. Normalization of NDI in bleomycin exposed lymphocytes that are previously treated with the decoction supports the protection mediated by the decoction against radiation induced damage.

Antioxidant activity is considered to be one of the major mechanisms by which many plants and phytochemicals are reported to offer radioprotection [40]. The potent antioxidant compounds present in the decoction may therefore be mainly responsible for the protective effect demonstrated by this herbal drug against bleomycin induced DNA damage in normal peripheral lymphocytes. The antioxidant potency of this polyherbal preparation is probably due to the collective contribution of antioxidant compounds present in the individual plant components used in its preparation. Thus, previous in vivo studies demonstrate that* N. sativa* seed oil can significantly reduce blood oxidative stress markers of rats exposed to a single dose of 6 Gy radiation [41]. Similarly, extracts of* H. indicus* that can protect against radiation induced strand breaks in plasmid DNA have also been shown to protect against oxidative stress induced lipid peroxidation [42]. Although these studies demonstrate the ability of the individual plants in the decoction to reduce oxidative stress, none of them have evaluated the ability of these plants to protect against cytogenetic damage induced by chemotherapy alone or in combination with radiotherapy.

Since the polyherbal decoction under current investigation consists of a number of active ingredients, previously reported multimechanistic approach of the decoction in counteracting tumour progression can reasonably be justified. Counteract to oxidative stress is one of such mechanisms that may render a resistance to chemotherapy or radiotherapy in malignant cells, but effective in protecting healthy cells during the said treatment modalities. However, such compromised anticancer activity of the decoction will be well compensated by the existence of the remaining anticancer mechanisms of the decoction.

In conclusion, the present investigation indicates that the decoction comprised of* N. sativa* seeds,* H. indicus* roots, and* S. glabra* rhizomes has the potential to protect against cytogenetic damage inflicted by bleomycin in human peripheral lymphocytes. Since the decoction under current investigation has previously been reported to mediate anticancer effect against certain malignancies, the observed radioprotective potential has a definite clinical advantage during its use among cancer patients. Thus, it could protect the patient against radiation induced cell damage while restricting tumour progression.

In the present investigation, the effect of the decoction was evaluated at a single preincubation period of 3 hrs. However, the in-cooperation of different preincubation periods may have a significant impact on the claimed radioprotective potential of the decoction under current investigation. Despite these limitations, the findings of the present study clearly indicate the broad functional spectrum of the decoction comprised of* N. sativa* seeds,* H. indicus* roots, and* S. glabra* rhizomes in cancer management.

## Figures and Tables

**Table 1:** Mean numbers of various chromosomal aberrations after damage induced by bleomycin (40 *µ*g/mL) in the presence or absence of the decoction (100–600 *µ*g/mL). DC, dicentric chromosome; AF, acentric fragments; ChB, chromatid breaks.

Experimental groups	Concentration (*μ*g/mL)	DC per 100 spreads	DC% inhibition	AF per 100 spreads	AF% inhibition	ChB per 100 spreads	ChB% inhibition
Negative control		0.037 ± 0.01		0.043 ± 0.01		0.063 ± 0.01	

Decoction control	Dec. 100 300 600	0.027 ± 0.01 0.026 ± 0.01 0.029 ± 0.02		0.057 ± 0.01 0.049 ± 0.02 0.054 ± 0.01		0.043 ± 0.02 0.041 ± 0.01 0.047 ± 0.02	

Bleomycin control	BLM 40	0.27 ± 0.04		0.36 ± 0.02		0.46 ± 0.04	

Test (decoction + bleomycin)	BLM 40 Dec. 100 300 600	0.17 ± 0.04 0.14 ± 0.03 0.16 ± 0.01	37.03 48.15 40.74	0.23 ± 0.02 0.19 ± 0.01 0.21 ± 0.02	36.11 47.23 41.67	0.34 ± 0.03 0.29 ± 0.02 0.33 ± 0.01	26.1 36.96 28.26

**Table 2:** The effect of the decoction at a dose range of 100–600 *µ*g/mL on micronuclei induction in vitro by bleomycin (40 *µ*g/mL) in cultured human lymphocytes. The values are mean ± SD per 1000 binucleated cells per individual of a sample population of 6 individuals. Nuclear division index (NDI) was expressed as mean ± SD for each experimental groups.

Experimental groups	Concentrations (*μ*g/mL)	MN/1000 BL (mean ± SD)	MN% inhibition	NDI (mean ± SD)
Negative control		0.00967 ± 0.00252		1.64333 ± 0.10786

Decoction control	Dec. 100 300 600	0.01167 ± 0.00252 0.00983 ± 0.00241 0.01187 ± 0.00213		1.66333 ± 0.10693

Bleomycin control	BLM 40	0.08833 ± 0.00603		1.47667 ± 0.10017

Test (decoction + bleomycin)	BLM 40 Dec. 100 300 600	0.025 ± 0.00265 0.021 ± 0.00231 0.024 ± 0.00243	71.69 76.23 72.83	1.6 ± 0.12166

**Table 3:** Number of lymphocytes bearing different number of *γ*-H2AX foci obtained from four (04) different experimental groups. Total count of 1000 lymphocytes was considered per each test group.

Experimental groups	Number of *γ*H2AX foci										
	0	1	2	3	4	5	6	7	8	9	10
Negative control	674	218	108	00	00	00	00	00	00	00	00
Decoction control (100 *µ*g/mL)	596	283	121	00	00	00	00	00	00	00	00
Bleomycin control (40 *µ*g/mL)	70	116	192	293	185	87	16	09	26	05	01
Test (decoction + bleomycin)	84	189	294	342	73	08	06	00	03	01	00
